# An invariants-based method for efficient identification of hybrid species from large-scale genomic data

**DOI:** 10.1186/s12862-019-1439-7

**Published:** 2019-05-30

**Authors:** Laura S. Kubatko, Julia Chifman

**Affiliations:** 1 Department of Statistics, The Ohio State University, Columbus, USA; 2 Department of Evolution, Ecology, and Organismal Biology, The Ohio State University, Columbus, USA; 3 Department of Mathematics and Statistics, American University, Washington, DC USA

**Keywords:** ABBA-BABA, Coalescence, Hybridization, Phylogenetic invariants

## Abstract

**Background:**

Coalescent-based species tree inference has become widely used in the analysis of genome-scale multilocus and SNP datasets when the goal is inference of a species-level phylogeny. However, numerous evolutionary processes are known to violate the assumptions of a coalescence-only model and complicate inference of the species tree. One such process is hybrid speciation, in which a species shares its ancestry with two distinct species. Although many methods have been proposed to detect hybrid speciation, only a few have considered both hybridization and coalescence in a unified framework, and these are generally limited to the setting in which putative hybrid species must be identified in advance.

**Results:**

Here we propose a method that can examine genome-scale data for a large number of taxa and detect those taxa that may have arisen via hybridization, as well as their potential “parental” taxa. The method is based on a model that considers both coalescence and hybridization together, and uses phylogenetic invariants to construct a test that scales well in terms of computational time for both the number of taxa and the amount of sequence data. We test the method using simulated data for up 20 taxa and 100,000bp, and find that the method accurately identifies both recent and ancient hybrid species in less than 30 s. We apply the method to two empirical datasets, one composed of *Sistrurus* rattlesnakes for which hybrid speciation is not supported by previous work, and one consisting of several species of *Heliconius* butterflies for which some evidence of hybrid speciation has been previously found.

**Conclusions:**

The proposed method is powerful for detecting hybridization for both recent and ancient hybridization events. The computations required can be carried out rapidly for a large number of sequences using genome-scale data, and the method is appropriate for both SNP and multilocus data.

## Background

Large-scale genomic data present many challenges in the inference of the evolutionary history of a collection of species. The most notable of these is the development of methods for inferring species-level phylogenetic relationships from multiple gene alignments that simultaneously incorporate the evolutionary processes that are known to contribute to variability in histories for the individual genes. Two important processes are incomplete lineage sorting (ILS) and hybridization [1]. ILS results when two gene copies fail to coalesce in the most recent ancestral population and is commonly modeled by the coalescent process, which provides a link between the species tree and the gene trees that represent the phylogenetic history for each gene [2–4]. In particular, multispecies coalescent theory models probabilities of rooted gene tree topologies within a given rooted species tree topology and has been used to derive the various probability distributions on gene trees given a particular species tree [5–10]. To date, many methods have been proposed for estimation of species phylogeny from multi-locus data based on the coalescent process (e.g., BEST [11], *BEAST [12], STEM [13], MP-EST [14], SNAPP [15], SVDquartets [16] (now implemented in PAUP* [17]), ASTRAL [18], among others).

Hybridization is another evolutionary process that can cause variability in gene trees within the containing species tree. It generally refers to the interbreeding of individuals from distinct populations, resulting in the production of a hybrid species that shares genetic information with both parental species. Hybridization between distinct species can occur for many generations with fertile offspring, making it possible for a new species to be formed. If the hybridization does not result in the formation of a new lineage, the process is termed introgression or introgressive hybridization [19–28]. Despite the earlier belief that hybridization was rare, numerous recent studies have shown that hybrid speciation occurs in both plants and animals [27, 29–38]. Hybridization has been recognized as an important mechanism for the evolution of new species and recent estimates indicate that approximately 25% of plants and 10% of animals hybridize [26, 27, 27, 28, 39]. However, inference of hybridization cannot be based solely on observed gene tree variability since other processes (e.g., incomplete lineage sorting and gene duplication and loss) may contribute to disagreements in single-gene phylogenies [1].

Several models and methods have been developed to detect hybridization. Here we focus on methods specific to gene flow between species (hybridization) and not on methods that are concerned with gene flow within one species (admixture). One group of methods for detecting hybridization involves the identification and removal of hybrids prior to phylogenetic analysis, with the hybrids added to the inferred tree by connecting them to their parental species [40–42]. Joly et al. (2009) [43] developed a method and software (JML; [44]) for identifying introgressed sequences by proposing that for some hybridization events the minimum distance between two sequences will be smaller than for incomplete lineage sorting. Another test that was originally developed to test ancient admixture is based on a relative abundance of ABBA or BABA single nucleotide patterns that can be evaluated using Patterson’s D-statistic [45–47]. However, Eaton and Ree (2013) [48] noted that Patterson’s D-statistic does not utilize all the information from incongruent allele patterns in multiple taxa and proposed an extension to the method, which they termed partitioned D-statistic. Meng and Kubatko (2009) [49] proposed a model for detecting hybridization under the coalescent model and used both a maximum likelihood and a Bayesian framework for inference. An extension to that model was later provided by Kubatko (2009) [50] by utilizing gene tree densities for inference. Yu et al. (2014) [51] also proposed a likelihood method that accounts for both reticulate evolutionary events and incomplete lineage sorting by providing methods for computing the likelihood of a phylogenetic network under the coalescent model. This method, as well as some earlier variations of it, is implemented in the software PhyloNet [52].

In this paper we develop a method for detecting and quantifying the extent of hybridization using a coalescent-based model that is fast and accurate. At the heart of our method are special relations called *phylogenetic invariants*, which are functions (usually polynomials) in the site pattern probabilities that evaluate to zero on any probability distribution that is consistent with the tree topology and associated model. Invariants have been introduced by Cavender and Felsenstein (1987) [53] and Lake (1987) [54] as a means for phylogenetic reconstruction, and have recently been gaining popularity for use in phylogenetic tree inference [16, 55, 56]. Here we propose using a ratio between two linear invariants in site pattern probabilities to develop statistics that accurately identify hybrid taxa. Because these statistics are functions of site pattern probabilities across multi-locus or SNP data, they can be rapidly computed. In addition, we can derive the mean, variance, and asymptotic distribution of these invariants, enabling development of a hypothesis test for hybridization when the number of sites is large. We begin by giving the theoretical details of our model, and then evaluate the performance of several possible invariants-based statistics for four-taxon trees using simulation. The best-performing of these statistics, which we call the Hils statistic, is then evaluated for larger trees using simulation, with hybridization events at various “depths” of the tree (i.e., hybridization between tip species and hybridization between ancestral species). Finally, we apply our method to several empirical data sets, including the *Sistrurus* rattlesnakes and *Heliconius* butterflies.

## Results

### A Coalescent-based Model for Hybridization

**Fig. 1:**
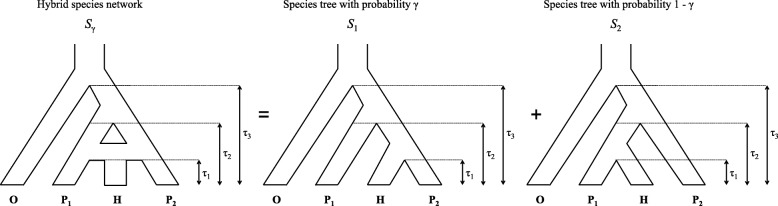
Coalescent model with hybridization. Model for the species-level relationships among four taxa under the coalescent model with hybridization. Here taxon *H* is a hybrid of taxa *P*_1_ and *P*_2_

We consider here the model originally proposed by Meng and Kubatko (2009) in which data arise along a phylogenetic species tree via an evolutionary process that allows for the possibility of both hybridization and incomplete lineage sorting, as modeled by the coalescent process. Hybridization cannot be modeled by a bifurcating phylogenetic tree, thus it is common to represent hybridization on a phylogeny by a horizontal line connecting two lineages of an otherwise-bifurcating phylogeny (Fig. 1 the leftmost panel). This network represents the evolutionary history of the species as a whole, and depicts a hybrid origin for taxon H. We refer to species *H* as the hybrid species, and to species *P*_1_ and *P*_2_ as the parental species. The times labeled by *τ*_*i*_ are speciation times, and in general we refer to the species network *S*_*γ*_ together with its vector of speciation times ***τ*** by (*S*_*γ*_,***τ***). The data arising along this phylogenetic species network are a collection of site patterns. Letting *X*_*Y*_∈{*A*,*C*,*G*,*T*} denote the nucleotide observed for species *Y* at a specific location in the DNA sequence, we define a site pattern $\mathbf {X} = X_{O} X_{P_{1}} X_{H} X_{P_{2}}\phantom {\dot {i}\!}$ as an assignment of nucleotides to all species. We represent the site pattern probability on the species network (*S*_*γ*_,***τ***) for a particular observation *ijkl* at the tips of the network by

1 $$ p_{ijkl | (S_{\gamma},\boldsymbol{\tau})} = P(X_{O}=i, X_{P_{1}}=j, X_{H}=k, X_{P_{2}}=l | (S_{\gamma},\boldsymbol{\tau}))  $$

for *i*,*j*,*k*,*l*∈{*A*,*C*,*G*,*T*}.

Our model defines the probability distribution on the space of all 4^4^=256 site patterns under a model that allows both ILS and hybridization via a three-stage process. First, the hybrid species is assigned one of its two putative parents, with probability *γ* of selecting parental species *P*_2_ and probability 1−*γ* of selecting parental species *P*_1_ (resulting in trees *S*_1_ and *S*_2_ in Fig. 1 being the “parental species trees”, respectively). Next, a gene tree is generated along the parental species tree from step 1 through the standard coalescent process (see, e.g., [2–5, 7, 9, 57, 58]). Finally, a site pattern is generated along the gene tree from step 2 according to one of the standard Markov substitution models (e.g., the GTR+I+ *Γ* model [59] or one of its sub-models). Combining steps 2 and 3, we see that the probability for site pattern *ijkl* for a given species tree *S*_*i*_, *i*∈{1,2}, is given by

$$ p_{ijkl | (S_{i},\boldsymbol{\tau})} = \sum\limits_{G} \int_{\mathbf{t}} p_{ijkl | (G, \mathbf{t})} f((G, \mathbf{t}) | (S_{i},\boldsymbol{\tau})) d \mathbf{t}, $$

where (*G*,**t**) represents a gene tree with topology *G* and branch lengths **t**, *p*_*ijkl*|(*G*,**t**)_ is the probability of the particular observation *ijkl* at the tips of gene tree (*G*,**t**), and *f*((*G*,**t**)|(*S*_*i*_,***τ***)) is the joint density of (*G*,**t**) conditional on the species tree (*S*_*i*_,***τ***). A full description of the computations required for this model are given in Chifman and Kubatko (2015) [60], and we do not review them here. Finally, we write the site pattern probability on a hybrid species network as

2 $$ p_{ijkl | (S_{\gamma},\boldsymbol{\tau})} = \gamma p_{ijkl | (S_{1}, \boldsymbol{\tau})} + (1-\gamma) p_{ijkl | (S_{2}, \boldsymbol{\tau})}.  $$

For our purposes, it suffices to view the collection of site patterns observed in an empirical data set as a sample of observations from the probability distribution defined by the $\{p_{ijkl | (S_{\gamma },\boldsymbol {\tau })} | i,j,k,l \in \{A,C,G,T\} \}$. We call data generated in this way “coalescent independent sites” and refer to this model as the “coalescent independent sites model”.

Let *N*_**X**_ be the number of sites with site pattern **X** observed in a sample of *N* sites generated from hybrid species network (*S*_*γ*_,***τ***) under this coalescent-with-hybridization model. Define $\mathbf {p} = (p_{AAAA|(S_{\gamma },\boldsymbol {\tau })}, p_{AAAC|(S_{\gamma },\boldsymbol {\tau })}, \ldots, p_{TTTT|(S_{\gamma },\boldsymbol {\tau })})$ and $\hat {\mathbf {p}} = (\hat {p}_{AAAA}, \hat {p}_{AAAC}, \ldots,\hat {p}_{TTTT})$, where $\hat {p}_{\mathbf {X}} = \frac {N_{\mathbf {X}}}{N}$. The vector $N\hat {\mathbf {p}}$ then gives the observed counts of the 256 possible site patterns in the sample, and thus

3 $$ N\hat{\mathbf{p}} \sim \textup{Multinomial}(N; \mathbf{p}).  $$

When *N* is large, the $\hat {p}_{\mathbf {X}}$ are approximately normally distributed, and thus the sampling distributions of statistics based on the $\hat {p}_{\mathbf {X}}$ can be derived. We next describe how these ideas can be used to build tests for hybridization.

### Invariants-based Hypothesis Tests for Hybridization

As mentioned in the Introduction, our tests are based on phylogenetic invariants, which are polynomials in the site patterns that evaluate to zero on one tree topology but do not evaluate to zero for at least one tree of a different topology.

Consider four linear relationships that arise on the hybrid phylogenetic species network (*S*_*γ*_,***τ***) as described in the previous section:

$$\begin{array}{*{20}l} f_{1} &=p_{iijj| (S_{\gamma},\boldsymbol{\tau})} - p_{ijij| (S_{\gamma},\boldsymbol{\tau})}, & f_{3} &= p_{ijii| (S_{\gamma},\boldsymbol{\tau})} - p_{iiji| (S_{\gamma}, \boldsymbol{\tau})}, \\ f_{2} &=p_{ijji | (S_{\gamma},\boldsymbol{\tau})} - p_{ijij| (S_{\gamma},\boldsymbol{\tau})}, & f_{4} &= p_{iiij| (S_{\gamma},\boldsymbol{\tau})} - p_{iiji| (S_{\gamma},\boldsymbol{\tau})}, \end{array} $$

where *i*≠*j*∈{*A*,*C*,*G*,*T*}. It can be shown that *f*_2_ and *f*_4_ are zero when evaluated on site pattern probabilities that correspond to the species tree *S*_1_, while *f*_1_ and *f*_3_ are non-zero (see [60] for details). Similarly, *f*_1_ and *f*_3_ are zero when evaluated on site pattern probabilities that correspond to tree *S*_2_, while *f*_2_ and *f*_4_ are not. However, when the site pattern probabilities correspond to the species network (*S*_*γ*_,***τ***) with *γ*∈(0,1), none of the four linear relations are zero.

What is special about these functions is that their ratio is a function of *γ*∈(0,1):

4 $$ {{} \begin{aligned} \frac{f_{1}}{f_{2}}&=\frac{p_{iijj| (S_{\gamma},\boldsymbol{\tau})} - p_{ijij| (S_{\gamma},\boldsymbol{\tau})} }{p_{ijji | (S_{\gamma},\boldsymbol{\tau})} - p_{ijij| (S_{\gamma},\boldsymbol{\tau})} }\\ &=\frac{\gamma \left(p_{iijj| (S_{1}, \boldsymbol{\tau})}-p_{ijij| (S_{1}, \boldsymbol{\tau})}\right) + (1-\gamma) \left(p_{iijj | (S_{2}, \boldsymbol{\tau})}-p_{ijij| (S_{2}, \boldsymbol{\tau})}\right)}{\gamma \left(p_{ijji| (S_{1}, \boldsymbol{\tau})}-p_{ijij| (S_{1}, \boldsymbol{\tau})}\right) + (1-\gamma) \left(p_{ijji | (S_{2}, \boldsymbol{\tau})}-p_{ijij| (S_{2}, \boldsymbol{\tau})}\right)}\\ &=\frac{\gamma \left(p_{iijj| (S_{1}, \boldsymbol{\tau})}-p_{ijij| (S_{1}, \boldsymbol{\tau})}\right) + (1-\gamma)(0)}{\gamma (0)+(1-\gamma) \left(p_{ijji | (S_{2}, \boldsymbol{\tau})}-p_{ijij| (S_{2}, \boldsymbol{\tau})}\right)} \\ &=\frac{\gamma}{1-\gamma}. \end{aligned}}  $$

Notice that the last equality holds because $p_{ijji | (S_{2}, \boldsymbol {\tau })}-p_{ijij| (S_{2}, \boldsymbol {\tau })}=p_{iijj| (S_{1}, \boldsymbol {\tau })}-p_{ijij| (S_{1}, \boldsymbol {\tau })}$, which results from the symmetric roles of *P*_1_ and *P*_2_ leading to $p_{ijji | (S_{2}, \boldsymbol {\tau })} = p_{iijj| (S_{1}, \boldsymbol {\tau })}$ and $p_{ijij| (S_{2}, \boldsymbol {\tau })} = p_{ijij| (S_{1}, \boldsymbol {\tau })}$. A full explanation about linear relations under the coalescent model on species trees that satisfy the molecular clock is provided in Chifman and Kubatko (2015), Section 3.1 [60]. Using a similar argument we find that

5 $$ \frac{f_{3}}{f_{4}}=\frac{\gamma}{1-\gamma} \quad \text{and} \quad \frac{f_{1} + f_{3}}{f_{2} + f_{4}}=\frac{\gamma}{1-\gamma}.  $$

If we consider cumulative site pattern probabilities then the results in Eqs. (4) and (5) still hold. By a cumulative site pattern we mean, for example, $p_{ijji|(S_{\gamma }, \boldsymbol {\tau })} = {\sum \nolimits }_{x \neq y \in \{A, C, G, T\}} p_{xyyx| (S_{\gamma }, \boldsymbol {\tau })}$. Under the JC69 model [61], each of the terms in the sum will have the same value, regardless of the choice of *x* and *y*; under more complex models, these probabilities will vary depending on the particular *x* and *y*. We implement the JC69 version of the test here, though we use simulation to assess the performance under more complicated models.

Using the ratios in Eqs. (4) and (5) we construct formal significance tests of the following hypotheses:

$$H_{0}: \gamma = 0 \text{ vs.}\ H_{1}: \gamma > 0. $$

Here we consider the ratio $\frac {f_{1}}{f_{2}}$ to illustrate the procedure. First, we estimate this ratio using the site pattern probabilities observed in the sample,

6 $$ \frac{\hat{f}_{1}}{\hat{f}_{2}} = \frac{\hat{p}_{iijj} - \hat{p}_{ijij}}{\hat{p}_{ijji} - \hat{p}_{ijij} }.  $$

To use this estimator as a test statistic in a hypothesis test, we need the distribution of the statistic when the null hypothesis is true. We first consider distributional results for the numerator and denominator separately. Using standard results for the multinomial distribution, we have

7 $$\begin{array}{@{}rcl@{}} \mu_{f_{1}} & := & E(\hat{p}_{iijj} - \hat{p}_{ijij}) = p_{iijj| (S_{\gamma},\boldsymbol{\tau})} - p_{ijij| (S_{\gamma},\boldsymbol{\tau})},  \end{array} $$

8 $$\begin{array}{@{}rcl@{}} \mu_{f_{2}} & := & E(\hat{p}_{ijji} - \hat{p}_{ijij}) = p_{ijji | (S_{\gamma},\boldsymbol{\tau})} - p_{ijij| (S_{\gamma},\boldsymbol{\tau})}, \end{array} $$

9 $$\begin{array}{@{}rcl@{}} \sigma^{2}_{f_{1}} & := & Var(\hat{p}_{iijj} - \hat{p}_{ijij}) = \frac{1}{N}(p_{iijj| (S_{\gamma},\boldsymbol{\tau})}(1-p_{iijj| (S_{\gamma},\boldsymbol{\tau})})  \\ & &\!\!\!\!\!\!\!\!\!\!\! + p_{ijij| (S_{\gamma},\boldsymbol{\tau})}(1-p_{ijij| (S_{\gamma},\boldsymbol{\tau})}) + 2p_{iijj| (S_{\gamma},\boldsymbol{\tau})} p_{ijij| (S_{\gamma},\boldsymbol{\tau})}), \end{array} $$

10 $$\begin{array}{@{}rcl@{}} \sigma^{2}_{f_{2}} & := & Var(\hat{p}_{ijji} - \hat{p}_{ijij}) = \frac{1}{N} (p_{ijji | (S_{\gamma},\boldsymbol{\tau})}(1-p_{ijji | (S_{\gamma},\boldsymbol{\tau})}) \\ & & \!\!\!\!\!\!\!\!\!\!\!\!+ p_{ijij| (S_{\gamma},\boldsymbol{\tau})}(1-p_{ijij| (S_{\gamma},\boldsymbol{\tau})}) + 2p_{ijji | (S_{\gamma},\boldsymbol{\tau})} p_{ijij| (S_{\gamma},\boldsymbol{\tau})}), \end{array} $$

11 $$\begin{array}{@{}rcl@{}} \sigma_{f_{1},f_{2}} & := & cov(\hat{p}_{iijj} - \hat{p}_{ijij}, \hat{p}_{ijji} - \hat{p}_{ijij})  \\ & & = \frac{1}{N}(-p_{iijj| (S_{\gamma},\boldsymbol{\tau})} p_{ijji | (S_{\gamma},\boldsymbol{\tau})} + p_{iijj| (S_{\gamma},\boldsymbol{\tau})} p_{ijij| (S_{\gamma},\boldsymbol{\tau})}  \\ & & \!\!\!\!\!\!\!\!\!\!\!+ p_{ijji | (S_{\gamma},\boldsymbol{\tau})} p_{ijij| (S_{\gamma},\boldsymbol{\tau})} + p_{ijij| (S_{\gamma},\boldsymbol{\tau})}(1-p_{ijij| (S_{\gamma},\boldsymbol{\tau})})). \end{array} $$

Now, using the fact that when the sample size *N* is large we have $\hat {f}_{1} \sim N(\mu _{f_{1}}, \sigma ^{2}_{f_{1}})$ and $\hat {f_{2}} \sim N(\mu _{f_{2}}, \sigma ^{2}_{f_{2}})$, we apply the Geary-Hinkley transformation [62, 63] to the ratio $\frac {\hat {f}_{1}}{\hat {f}_{2}}$ to get

12 $$ \frac{\left(\mu_{f_{2}} \frac{\hat{f}_{1}}{\hat{f}_{2}} - \mu_{f_{1}}\right)}{\sqrt{\sigma_{f_{2}}^{2} \left(\frac{\hat{f}_{1}}{\hat{f}_{2}}\right)^{2}-2\sigma_{f_{1},f_{2}}\frac{\hat{f}_{1}}{\hat{f}_{2}}+\sigma_{f_{1}}^{2}}} \sim N(0,1).  $$

The terms in the denominator on the left-hand side of the above equation depend on several unknown quantities, which we estimate by substituting the observed site pattern frequencies into Eqs. (7) - (11). We also multiply the expression in Eq. (12) by $\frac {\hat {f_{2}}}{\mu _{f_{2}}}$ (which converges in probability to 1, and thus does not change the asymptotic distribution) to obtain the test statistic

13 $$ H := \frac{\hat{f}_{2}(\frac{\hat{f}_{1}}{\hat{f}_{2}} - \frac{\mu_{f_{1}}}{\mu_{f_{2}}})}{\sqrt{\hat{\sigma}_{f_{2}}^{2} (\frac{\hat{f}_{1}}{\hat{f}_{2}})^{2}-2\hat{\sigma}_{f_{1},f_{2}}\frac{\hat{f}_{1}}{\hat{f}_{2}}+\hat{\sigma}_{f_{1}}^{2}}}.  $$

We call the statistic *H* the Hils statistic, in honor of Professor Matthew H. Hils ^*a*^. Under the null hypothesis that *γ*=0, the term $\frac {\mu _{f_{1}}}{\mu _{f_{2}}}$ in the numerator of (13) is 0, and the hypothesis test can be carried out by comparing the observed value of the test statistic computed with $\frac {\mu _{f_{1}}}{\mu _{f_{2}}}=0$ to a standard normal distribution. Tests based on the ratios $\frac {f_{3}}{f_{4}}$ and $\frac {f_{1}+f_{3}}{f_{2}+f_{4}}$ can be derived analogously.

We note that *γ*=1 also implies the absence of hybridization, and thus our hypothesis test should consider this situation as well. In fact, the symmetry in the model in Fig. 1 means that this case is already covered by the test above. To see this, note that when *γ*=0, $\hat {f}_{1}$ is close to 0, and the hypothesis test will fail to reject the null hypothesis, as could be expected from inspection of Eq. (13). When *γ*=1, then $\hat {f}_{2}$ is close to 0. It is not obvious from Eq. (13) that the test statistic would be expected to be close to 0 in this case, but if one multiples both the numerator and the denominator of the test statistic in Eq. (13) by $\frac {\hat {f}_{2}}{\hat {f}_{1}}$, it can be observed that there is an equivalent version of the test statistic with $\hat {f}_{2}$, rather than $\hat {f}_{1}$, in the numerator. Note that our condition $\hat {p}_{ijij}>max\{\hat {p}_{iijj}, \hat {p}_{ijji} \}$ (see below) ensures that both $\hat {f}_{1}$ and $\hat {f}_{2}$ are positive. Thus the test given above is sufficient to test for hybridization with either *γ*=0 or *γ*=1.

### Extension to Larger Species Networks

The hypothesis test derived in the previous section deals with the case in which four taxa are specified, with one of the four taxa identified as the putative hybrid species. In many settings, however, primary interest is in searching over a large collection of species with the goal of identifying which species might have arisen via a process that involved hybridization at some point in the past. To address this, we consider a large collection of sequences, and suppose that an outgroup sequence can be identified. For each subset of four sequences consisting of three sequences plus the outgroup, we carry out the above test of hybridization for different assignments of the three ingroup sequences to the hybrid and parental taxa. Of the three possible choices for the hybrid taxon, we consider only two of those, eliminating from consideration the one for which $\hat {p}_{ijij}>max\{\hat {p}_{iijj}, \hat {p}_{ijji} \}$, since this implies that the two parental taxa are more closely related than either is to the putative hybrid. For a data set of *n*+1 sequences with one outgroup sequence, this results in $\binom {n}{3}\times 2$ hypothesis tests. To handle the issue of multiple comparisons, we use the Bonferroni correction, which is conservative in this case because the tests are correlated. Thus, if an overall *α*-level test is desired, we report significant evidence of hybridization when the *p*-value computed for a particular comparison is smaller than $\frac {\alpha }{ \binom {n}{3} \times 2}$.

The simulation design for each study is described in the “Methods” section and all code used to carry out the simulations and empirical analyses in this paper is available at https://github.com/lkubatko/HilsTest.

### Four-taxon simulation studies

**Fig. 2:**
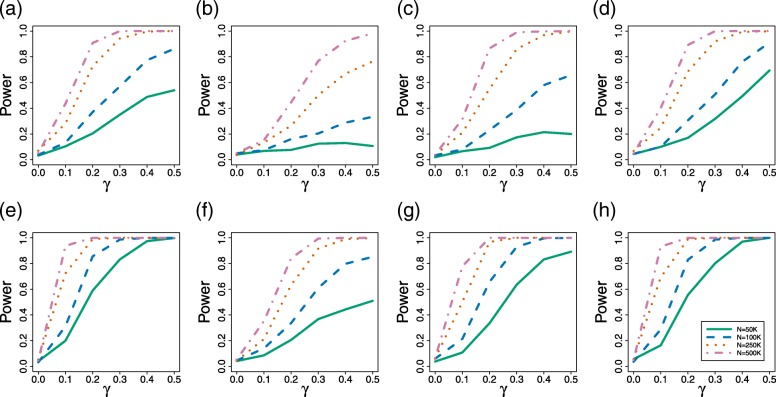
Power Plots. Results of the power simulations for the four-taxon hybrid species network in Fig. 1. Plots (**a**), (**b**), (**c**), and (**d**) correspond to data simulated for the “short” branch length setting, and plots (**e**), (**f**), (**g**), and (**h**) correspond to data simulated for the “long” branch length settings. Plots (**a**) and (**e**) give results for the test based on $\frac {f_{1}}{f_{2}}$; plots (**b**) and (**f**) give results for the test based $\frac {f_{3}}{f_{4}}$; plots (**c**) and (**g**) give results for the test based on $\frac {f_{1}+f_{3}}{f_{2}+f_{4}}$; and plots (**d**) and (**h**) give results for the ABBA-BABA test

Our results for the four-taxon simulation studies establish that the various tests behaved as we have expected (Fig. 2 and Table 1). First, in all of the cases considered, the power increases as the sample size increases, reaching near 100% when alignments of length 500,000bp were used for many of the simulation conditions (Fig. 2). Second, we note that as the value of *γ* increases from 0 (no hybridization) to 0.5 (equal contribution from both parental species), the power to detect hybridization increases as well, with near 100% power for the “long” branch length setting when *γ*≥0.3 for all three of the tests considered. Third, we note that all of the tests are more powerful for data simulated under the “long” branch length setting (Fig. 2e, f, g, and h) than for data generated under the “short” branch length setting (Fig. 2a, b, c, and d). Finally, we note that all tests appear to achieve the nominal 0.05 level when data are simulated under the null hypothesis (*γ*=0). The ABBA-BABA test (Fig. 2d and h) shows power similar to our test based on the ratio $\frac {f_{1}}{f_{2}}$ (Fig. 2a and e).

**Table 1:** Estimates of the parameter *γ* using the ratio $\frac {f_{1} }{ f_{2}}$ for data simulated on the four-taxon hybrid species network in Fig. 1 with the “short” and “long” branch lengths settings

	``Short" branch length				``Long" branch length							
***γ***	0	0.1	0.2	0.3	0.4	0.5	0	0.1	0.2	0.3	0.4	0.5
500K												
Mean	0.0052	0.0986	0.2012	0.2984	0.4029	0.5005	-0.0006	0.0990	0.1981	0.2993	0.3993	0.4994
SD	0.0604	0.0524	0.0484	0.0427	0.0388	0.0356	0.0281	0.0245	0.0241	0.0220	0.0209	0.0205
MSE	0.0037	0.0027	0.0023	0.0018	0.0015	0.0013	0.0008	0.0006	0.0006	0.0005	0.0004	0.0004
250K												
Mean	-0.0061	0.0945	0.2048	0.2989	0.3958	0.5009	-0.0003	0.1009	0.1971	0.2985	0.3979	0.5004
SD	0.1008	0.0816	0.0665	0.0602	0.0555	0.0546	0.0407	0.0367	0.0355	0.0317	0.0300	0.0286
MSE	0.0102	0.0067	0.0044	0.0036	0.0031	0.0030	0.0017	0.0013	0.0013	0.0010	0.0009	0.0008
100K												
Mean	-0.0121	0.0789	0.1808	0.2784	0.3976	0.5001	-0.0053	0.0926	0.1997	0.3012	0.3986	0.5013
SD	0.3792	0.1445	0.1301	0.1079	0.0987	0.0930	0.0660	0.0616	0.0545	0.0498	0.0486	0.0456
MSE	0.1439	0.0213	0.0173	0.0121	0.0097	0.0087	0.0044	0.0039	0.0030	0.0025	0.0024	0.0021
50K												
Mean	-0.0532	0.0157	0.0471	0.2542	0.4081	0.5282	-0.0152	0.0902	0.1955	0.2909	0.3983	0.4960
SD	0.5451	1.2607	1.4385	0.3020	0.8163	0.4126	0.1098	0.0884	0.0818	0.0793	0.0690	0.0653
MSE	0.2999	1.5964	2.0926	0.0933	0.6664	0.1710	0.0123	0.0079	0.0067	0.0064	0.0048	0.0043

One unexpected result of the simulations designed to address the power was that the test based on $\frac {f_{1}}{f_{2}}$ is more powerful than the tests based on $\frac {f_{3}}{f_{4}}$ and $\frac {f_{1}+f_{3}}{f_{2}+f_{4}}$. This is most likely due to the variance associated with estimating the various site pattern probabilities that contribute to each invariant. We return to this point in the discussion. Based on this observation, we report results for only the ratio $\frac {f_{1}}{f_{2}}$ in what follows.

The results of the four-taxon simulation studies designed to estimate *γ* using the ratio $\frac {f_{1}}{f_{2}}$ also matched our intuition about how the method should perform (Table 1). As the sample size increases, the estimates become closer to the true values used to generate the data, and the variance decreases as the sample size increases. In general, the estimates obtained from the “long” branch length setting are slightly better than those obtained from data generated under the “short” branch length setting. Overall, the method seems to provide very reasonable estimates of *γ*.

**Fig. 3:**
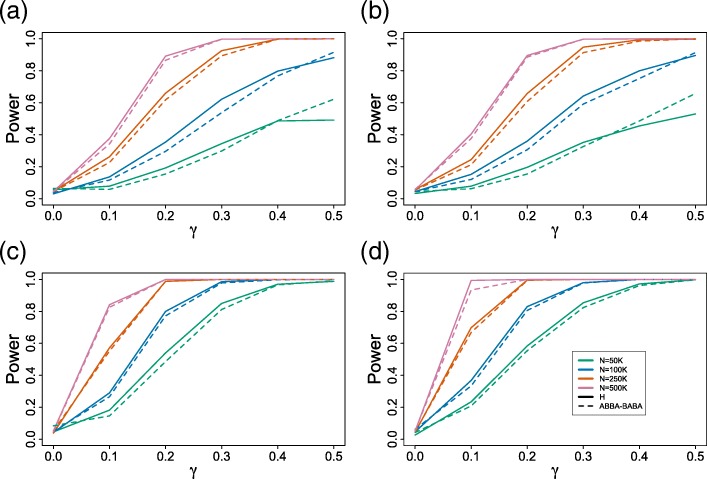
Power Plots. Results of the second set of power simulations for the four-taxon hybrid species network in Fig. 1. Plots (**a**) and (**b**) correspond to data simulated for the “short” branch length setting, and plots (**c**) and (**d**) correspond to data simulated for the “long” branch length setting. Plots (**a**) and (**c**) correspond to data simulated under a model species tree in which the length of the branch leading to species *P*1 is doubled; plots (**b**) and (**d**) correspond to data simulated under a model species tree in which the length of the branch leading to species *H* is doubled. In each plot, the solid lines show results for the Hils test, while the dotted lines show results for the ABBA-BABA test

The results of the second set of simulation studies are shown in Fig. 3. The results are in general consistent with the results of the first simulation study. In particular, the power increases as *γ* gets closer to 0.5 and as the sample size increases, and both tests are more powerful when the branch lengths are longer. The Hils test is slightly more powerful than the ABBA-BABA test over most of the simulation conditions examined, but from a practical viewpoint, little difference in performance of the two methods would be expected. While both tests show some decrease in power resulting from the violation of the molecular clock, both still perform well, particularly with sufficient data, suggesting that these methods have some degree of robustness to violation of the assumption of a molecular clock.

### Simulation studies for larger species networks

**Fig. 4:**
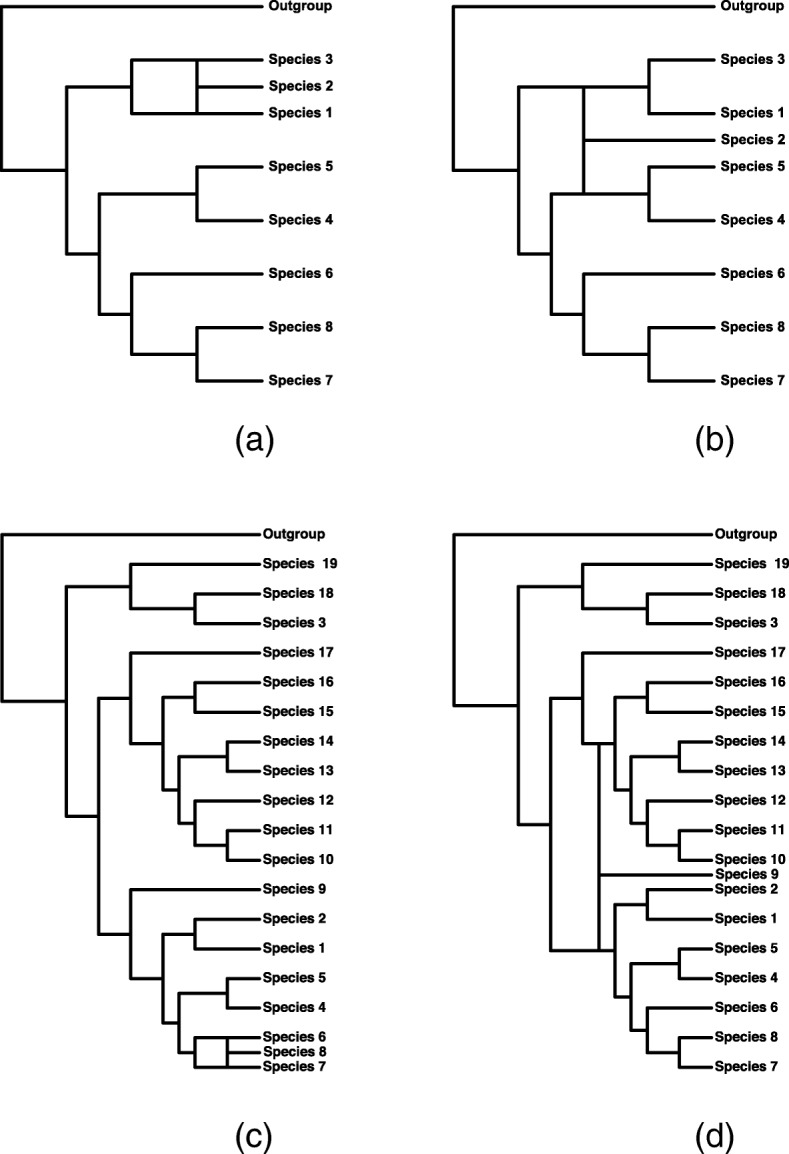
Trees for Simulation Study. Model networks with 9 and 20 taxa and with either shallow or deep hybridization used for the simulation studies. **a** 9-taxon shallow hybridization **b** 9-taxon deep hybridization **c** 20-taxon shallow hybridization **d** 20-taxon deep hybridization

For the 9-taxon simulations (Fig. 4 and Table 2), we note first that for data generated under the coalescent independent sites model, when *γ*=0 approximately 5% of the data sets give significant results, and thus the test appears to attain the desired significance level in this case. For the multilocus data sets, however, the type I error rate is larger than the specified 0.05 level, and thus the test appears to reject the null hypothesis more often than it should. When *γ*>0, we see that the test is powerful for both the shallow and the deep hybridization events and for both types of data, with the power above 90% in both cases when *γ*≥0.2. Furthermore, the test almost always selects the correct assignment of hybrid and parental taxa, with the proportion of times that this is exclusively generated increasing toward 100% as *γ* increases for the coalescent independent sites data. One observation we made that is not reflected in the results in Table 2 is that for data simulated from the network involving the deep hybridization event, many sets appear as significant when some true relationship is detected. For example, it is common to have the hybrid correctly assigned, but the parental species assigned as belonging to a taxon from the sister clade of the true parent. This is especially true for the multilocus data sets with the deep hybridization event. In other words, this test is good at picking out the hybrid taxon, but not as good at unambiguously picking out its parents when the hybridization event occurs deeper in the network. This was not the case for the shallow event, where it often got exactly the correct relationships and only those in most cases.

**Table 2:** Results of the simulation study for 9 taxa

	Coalescent Independent Sites				Multi-locus Data							
	Shallow Hybridization			Deep Hybridization			Shallow Hybridization			Deep Hybridization		
*γ*	False	True	True	False	True	True	False	True	True	False	True	True
	Pos.	Pos.	Sets	Pos.	Pos.	Sets	Pos.	Pos.	Sets	Pos.	Pos.	Sets
0	0.064	–	–	0.048	–	–	0.216	–	–	0.224	–	–
0.1	0.024	0.384	0.192	0.024	0.664	0.520	0.176	0.424	0.312	0.088	0.719	0.552
0.2	0.032	0.968	0.920	0.000	0.952	0.944	0.000	0.968	0.864	0.000	1.000	0.896
0.3	0.032	0.976	0.976	0.000	0.976	0.976	0.000	1.000	0.968	0.000	1.000	1.000
0.4	0.008	0.976	0.144	0.000	1.000	0.448	0.000	1.000	0.248	0.000	1.000	1.000
0.5	0.016	0.960	0.000	0.000	0.952	0.000	0.000	1.000	0.000	0.000	1.000	1.000

The columns labeled “False Pos.” refer to the proportion of data sets for which a triplet of taxa were incorrectly identified as involving a hybridization event (false positives); the columns labeled “True Pos.” refer to the proportion of data sets for which the correct triplet of taxa involving the hybridization event was identified *and* the hybrid taxon was correctly identified (true positives); and the columns labeled “True Sets” refer to the proportion of data sets for which the correct triplet of taxa was identified but the hybrid taxa was specified incorrectly. It is possible that *both* the correct triplet with the hybrid correctly specified and the correct triplet with the hybrid misspecified are identified as statistically significant in the analysis. We tally these separately because in the case of empirical data it would be ambiguous as to which is the hybrid taxon. For our simulated data, all data sets for which the true set was significant also had the triplet with the correct hybrid assignment found to be significant, and thus this proportion is always a fraction of the proportion of true positives

The results for the 20-taxon networks are largely the same (Fig. 4 and Table 3). The test still demonstrates good power to detect the hybridization event, though the power does not rise above 90% for all settings until *γ*≥0.3, rather than 0.2 as in the 9-taxon case. In addition, the proportion of data sets with “Correct Sets” decreases for the shallow hybridization events in this case, meaning that when a hybridization event is identified, it nearly always involved correct identification of which species was the hybrid and which were the parental species. Though there is a hint of an elevated type I error rate when multilocus data were simulated, the problem is not as dramatic as in the 9-taxon case. Overall, the method maintains its good ability to detect hybrid species.

**Table 3:** Results of the simulation study for 20 taxa

	Coalescent Independent Sites				Multi-locus Data							
	Shallow Hybridization			Deep Hybridization			Shallow Hybridization			Deep Hybridization		
*γ*	False	True	True	False	True	True	False	True	True	False	True	True
	Pos.	Pos.	Sets	Pos.	Pos.	Sets	Pos.	Pos.	Sets	Pos.	Pos.	Sets
0	0.048	–	–	0.040	–	–	0.064	–	–	0.072	–	–
0.1	0.008	0.072	0.008	0.000	1.000	0.240	0.112	0.008	0.064	0.008	0.648	0.352
0.2	0.000	0.704	0.096	0.000	0.936	0.936	0.016	0.688	0.160	0.000	0.984	0.960
0.3	0.000	0.952	0.080	0.000	0.928	0.928	0.000	1.000	0.168	0.000	1.000	1.000
0.4	0.000	0.960	0.000	0.000	0.968	0.968	0.000	1.000	0.000	0.000	1.000	0.976
0.5	0.000	0.952	0.000	0.000	0.984	0.928	0.000	1.000	0.000	0.000	1.000	0.896

Column headings are as in Table 2

### Empirical data: Sistrurus rattlesnakes

Recall that this dataset contains two species, each containing three subspecies, as well as two outgroup species, for a total of eight tips in the species phylogeny of interest. When analyzing empirical data of this nature, for which several individuals are sampled within each species, our main interest will be in detecting individuals that show evidence of hybrid origin from parental individuals that are members of two different species. The current version of our software will output the test statistic for all assignments of hybrid and parental taxa for a given outgroup, but this output can easily be examined to consider only the comparisons of interest. For the rattlesnake data for a particular choice of outgroup, we can consider all choices of one individual allele from each of three subspecies, and for each such choice, one individual will be assigned to be the hybrid and the other two assigned to be the parental taxa. For example, we can select one Sca individual, one Sce individual, and one Sct individual, and carry out the Hils test for each possible choice of hybrid among these three. Thus, for our particular data set consisting of 18 Sca alleles, 8 Sce alleles, 10 Sct alleles, 2 Smm alleles, 6 Smb alleles, and 4 Sms alleles, there will be ${\sum \nolimits }_{n_{i} \in \{0,1\}, \sum \nolimits n_{i}=3} \binom {18}{n_{1}}\binom {8}{n_{2}}\binom {10}{n_{3}}\binom {2}{n_{4}}\binom {6}{n_{5}}\binom {4}{n_{6}} = 7,840$ possible choices of three alleles, and two test statistics will be computed on each, resulting in 2∗7840=15,680 possible comparisons for each choice of outgroup sequence. We carry out the Bonferroni correction within the analysis for each outgroup, and thus each comparison uses significance level *α*=0.05/15680=0.0000032.

An additional practical issue that arose with our empirical data but was not observed with simulated data was that for some choices of three alleles, one or more of the site pattern frequencies *p*_*iijj*_,*p*_*ijij*_, and *p*_*ijji*_ was observed to be 0. To correct for this, we added a small count (0.005) to each observed site pattern count in all cases before computing estimated site pattern frequencies and carrying out the test. With this modification, we find no evidence of hybrid origin for any of the sequences with any choice of outgroup sequence, consistent with other analyses in this group [64, 65].

### Empirical data: Heliconius butterflies

This dataset consists of 3 species with 4 individuals sampled per species, plus an outgroup. Thus, the number of comparisons of interest is 4·4·4·2=128 and the Bonferroni-corrected level of the tests is 0.05/128=0.00039. The analysis of all possible hybrid/parental combinations for the alignment of length ≈ 248 million bp took 16 min on a 2 × Quad Core Xeon E5520 / 2.26GHz / 32GB desktop linux machine. All comparisons were statistically significant at the 0.00039 level. This result is not surprising, given the previous evidence of hybridization as described in Martin et al. (2013), and given the large sample size. What is interesting, however, is the strength of the evidence for hybridization. For example, across all comparisons in which an *H. m. rosina* individual was specified as the hybrid, the smallest test statistic was 172.6143, indicating overwhelming evidence for hybridization (recall that we are comparing to a standard normal distribution). In contrast, when one of the other species was identified as the hybrid and *H. m. rosina* was (incorrectly) identified as a parental taxon, the values of the test statistic ranged from ∼ 55 to 76, again indicating strongly significant deviation from the expected patterns under no gene flow, but not as strong as the case in which the hybrid is correctly identified as *H. m. rosina*. Overall, these results are in agreement with the work of Martin et al. (2013) on this group, and demonstrate the utility of our method in rapidly identifying hybrid taxa from genome-scale data.

## Discussion

We have proposed a method for detecting hybrid species using a model of hybrid speciation that incorporates coalescent stochasticity. The test is based on observed site pattern frequencies, which leads to several convenient properties. First, the computations required for the test can be carried out very rapidly, as all that is required is to obtain counts of observed site pattern frequencies for four taxa of interest. This computation is so rapid that there are essentially no limits on the length of sequences that can be handled by the method, and it is thus appropriate for genome-scale data. Second, observed site pattern frequencies arise from a multinomial distribution under the coalescent hybridization model used here, which allows derivation of the asymptotic distribution of the estimators of the site pattern frequencies. This ultimately leads to a null distribution for testing the hypothesis of interest that is asymptotically normally distributed which provides a straightforward test of the hypothesis of interest. Finally, we note that our method is derived under the assumption that each site has its own underlying gene tree, an experimental design that we call “coalescent independent sites”. The method is thus clearly appropriate for genome-wide SNP data, whether biallelic or not. We argue that the method is also appropriate for multilocus data, in that as the number of loci becomes large and provided that alignment lengths are not biased toward certain gene tree topologies, the proportion of sites observed from a particular gene tree will approach the proportion expected under the coalescent independent sites model. We thus carry out simulations for both multilocus and coalescent independent sites data, and we test our method on an empirical multilocus dataset.

Our simulations show that the method is powerful for detecting hybridization for both recent and ancient hybridization events, although for ancient hybridization events it may be more difficult to pinpoint the precise parental species for the detected hybrids. In addition, the proportional contribution of the two parental species to the genome of the hybrid species can be estimated accurately and unbiasedly. The simulations also show that the method scales extremely well: for 20-taxon networks with 100,000 sites, computations can be completed in less than 30 s, while for a dataset with 13 sequences and over 248 million sites, the analysis took less than 20 min on an older desktop linux machine. While these analyses demonstrate that sequence length is not a computationally-limiting factor, they also suggest that larger numbers of taxa will be similarly unproblematic. Although adding taxa increases the number of hypothesis tests to be carried out, these are each done very rapidly (e.g., for 20 taxa, there are over 2200 tests being done in less than 30 s), and they could easily be carried out on separate processors, if necessary. To the extent of our knowledge, this method is thus the only technique available for exploratory hybrid identification for large numbers of sequences using genome-scale data.

The method is based on phylogenetic invariants, and we note that the particular choice of invariants used here was somewhat arbitrary. Indeed, the ABBA-BABA test [45–47] is based on the difference of ABBA and BABA patterns similar to our invariant *f*_2_ and it too is useful in detecting hybridization. However their statistic is normalized by the total number of observations whereas our method is based on the ratio of two linear invariants leading to a function that depends only on the mixing parameter *γ*. Based on this crucial observation we were able to derive the Hils statistic for accurate detection of hybridization. We have also noticed that the ratio between *f*_3_ and *f*_4_ was not as powerful, thus it is possible that other invariants may be identified that work as well or better than the ones we have chosen here. It is also possible that invariants that operate on more than four taxa at a time could be determined, with potential improvements in the localization of hybrid and parental taxa for more ancient hybridization events. There is also a possibility that a set of linear invariants specific to species trees under the coalescent exists and can be classified, and if such a set exists, these species invariants may improve the performance. We suggest that exploring these directions is appealing, as site pattern-based methods provide the possibility of both rapid computation and convenient asymptotic distributions, making them suitable for processing the large genome-scale datasets that are becoming increasingly available. In fact, the performance of these methods improves with sequence length, since site pattern probabilities can be more accurately estimated, with little associated computational cost.

## Conclusions

Classification of organisms and estimation of their phylogenetic relationships is central to many areas of biological research, but inference of these relationships comes with several challenges. Most notable are computational challenges arising from the abundance of available DNA sequence data and the need to model organismal evolution at two distinct levels – individual genes, and species as a whole, where the evolutionary histories of genes are constrained by the evolutionary history of the species. Additionally, several processes, such as incomplete lineage sorting (deep coalescence), hybridization, horizontal gene transfer, and gene duplication and loss, lead to the potential for incongruence in the evolutionary histories of the individual genes. The multispecies coalescent is commonly used to model incomplete lineage sorting and provides a model for the generation of gene trees within the containing species tree. We used this model to develop a method for detecting species that have arisen via hybridization and for quantifying the extent of hybridization in a formal statistical framework. We demonstrated the performance of our method using both simulated and empirical data. Our method is capable of processing genome-scale sequence datasets consisting of many taxa in a computationally efficient manner, thus providing researchers with an effective exploratory tool for hybrid identification.

## Methods

### Simulation Studies

#### Four-taxon species networks

Our first set of simulation studies involves assessing the level and the power of the tests under various choices of the sample size, species trees branch lengths, and value of *γ* for four-taxon trees. We used a custom python program (available at https://github.com/lkubatko/HilsTest) to simulate gene trees from the two parental species trees in Fig. 1 with *γ* values of 0, 0.1, 0.2, 0.3, 0.4, and 0.5 and for two sets of speciation times: *τ*_1_=0.25,*τ*_2_=0.5,*τ*_3_=1.0 (the “short” setting) and *τ*_1_=0.5,*τ*_2_=1.0,*τ*_3_=2.0 (the “long” setting). For each setting, we simulated *N*=50,000,100,000,250,000 and 500,000 coalescent independent sites under the GTR+I+ *Γ* model using Seq-Gen [66](Seq-Gen options: -mGTR -r 1.0 0.2 10.0 0.75 3.2 1.6 -f 0.15 0.35 0.15 0.35 -i 0.2 -a 5.0 -g 3). For each parameter setting, we generated 500 replicate data sets.

For each simulated data set, we tested the null hypothesis that *γ*=0 using the test statistics corresponding to the ratios in Eqs. (4) and (5) at level *α*=0.05. We also applied the ABBA-BABA test [46]. We estimate the power of each test as the proportion of the 500 replicates for which the null hypothesis was rejected (when *γ*=0, this gives an estimate of the level of the test). We also considered using each of the statistics to estimate the true hybridization parameter, *γ*. We report the mean of the estimated *γ* values, as well as the standard deviation and the mean squared error, for each parameter setting.

To evaluate the sensitivity of our test to the assumption of a molecular clock, we carried out a second set of simulations using model trees that violated the clock assumption. We considered violating the molecular clock in two ways. First, we extended the branch leading to species *P*1 by doubling its length, for both the short and the long branch length settings described above. Second, we extended the branch leading to the hybrid species by doubling its length, again for both branch length settings. As in the first set of simulation studies, we evaluate the power of our test and compare its performance to the ABBA-BABA test. Here, however, we consider only the Hils test based on the ratio $\frac {f_{1}}{f_{2}}$, since this statistic showed superior performance in the first set of simulations.

#### Larger species networks

To examine the performance of our method for larger taxon samples, we considered networks containing 8 species and an outgroup, and networks containing 19 species and an outgroup. We also considered both recent hybridization and more ancient hybridization in each case (Fig. 4). For each model network, we generated 125 data sets containing 100,000 coalescent independent sites for *γ*=0,0.1,0.2,0.3,0.4, and 0.5 as follows. First, 100000*γ* gene trees were generated from the species tree formed by connecting the hybrid taxon to the “left” parental lineage, and 100000(1−*γ*) gene trees were generated from the species tree formed by connecting the hybrid taxon to the “right” parental lineage. For each gene tree, one coalescent independent site was generated using Seq-Gen [66] under the GTR+I+ *Γ* model (Seq-Gen options: -mGTR -r 1.0 0.2 10.0 0.75 3.2 1.6 -f 0.15 0.35 0.15 0.35 -i 0.2 -a 5.0 -g 3). Each simulated data set was then given to our program with the outgroup specified, and the Hils statistic was computed for each possible combination of parents and hybrids. A cut-off for significance was determined using a Bonferroni correction with base level *α*=0.05, and the putative hybrid and parents were reported for any statistic whose *p*-value fell below *α*/*M*, where *M* was the total number of comparisons. We summarized results by counting the number of “True Positives” (data sets for which the true hybrid and parental taxa are correctly identified), “True Sets” (data sets for which the true hybrid and parental taxa are identified, but their assignment to which is the hybrid and which are the parental taxa is ambiguous), and “False Positives” (data sets for which an incorrect set of taxa are identified as being subject to hybridization).

Because many of the genome-scale datasets being generated today are multilocus datasets (rather than being generated under the coalescent independent sites model used here), we also simulated data under multilocus n. These simulations proceeded exactly as described above, except that rather than simulating 100,000 coalescent independent sites, we simulated 1000 genes each of length 100bp. This choice was made to mimic the short read lengths generated by next-gen sequencing methods. We summarized these results in the same manner as described above. We justify application of our methodology to multilocus data in the Discussion section.

### Empirical examples

We have also explored the performance of our method on two empirical data sets; the *Sistrurus* rattlesnakes and *Heliconius* butterflies. The *Sistrurus* rattlesnakes are found across North America and are currently classified into two species, *Sistrurus catenatus* and *S. miliarius*, each with three putative subspecies. The dataset consists of 19 genes sampled from 26 rattlesnakes: 18 individuals within the species *Sistrurus catenatus* (with subspecies *S. c. catenatus* (Sca, 9 individuals), *S. c. edwardsii* (Sce, 4 individuals), and *S. c. tergeminus* (Sct, 5 individuals)); six within species *Sistrurus miliarius* (with subspecies *S. m. miliarius* (Smm, 1 individual), *S. m. barbouri* (Smb, 3 individuals), and *S. m. streckeri* (Sms, 2 individuals)); and two outgroup species, *Agkistrodon contortrix* and *A. piscivorus*. These data were originally analyzed by [67] to determine species-level phylogenetic relationships. Prior to this analysis, the sequences were computationally phased, resulting in 52 sequences and 8,466 aligned nucleotide positions (data are available at TreeBase ID 11174). These data have been subsequently reanalyzed in several ways. For example, [16] used different methodology to infer the species phylogeny, and found agreement with the original analysis of Kubatko et al. (2011). Gerard et al. (2011) used a subset of the data to examine whether several specimens collected in Missouri and assigned to subspecies *S. c. catenatus* were actually hybrid species. They did not find evidence of hybridization, in agreement with other results using different data [64].

The *Heliconius* butterflies are a diverse group of tropical butterflies in the family *Heliconii* that are found throughout the southern United States and in Central and South America. We consider the study of Martin et al. (2013) [68] in which genome-scale data for 31 individuals from seven distinct species were collected and evidence for gene flow between various species was assessed. We examine a subset of these data consisting of four individuals from each of the species *Heliconius cydno*, *H. melpomene rosina*, and *H. m. melpomene*, as well as one individual from the outgroup species *H. hecale*. Martin et al. (2013) found evidence that *H. m. rosina* is a hybrid of *H. m. melpomene* and *H. cydno*. We obtained the aligned genome-wide data from the complete study of Martin et al. (2013) from Dryad (http://datadryad.org/resource/doi:10.5061/dryad.dk712) [69], and extracted the 13 sequences of interest. The resulting aligned sequences consisted of 248,822,400 base pairs.

## Abbreviations

ABBA-BABA: Patterson’s D-statistic to test ancient admixture
ASTRAL: Accurate Species TRee ALgorithm
BEAST: Bayesian Evolutionary Analysis Sampling Trees
BEST: Bayesian Estimation of Species Trees
GTR+I+ *Γ*: General time-reversible model of Tavaré1986 with site-specific rate variation, and invariable sites
ILS: Incomplete Lineage Sorting
JC69: the Jukes and Cantor 1969 model of DNA evolution
JML: Testing hybridization from species trees
MP-EST: Maximum Pseudo-likelihood for Estimating Species Trees
PAUP*: Phylogenetic Analysis Using Parsimony *and other methods Seq-Gen: Sequence-Generator
SNAP: SNP and AFLP Package for Phylogenetic analysis
SNP: Single Nucleotide Polymorphism
SVDquartets: Singular Value Decomposition Scores for Species Quartets
